# Dose-Limiting Toxicities and the Maximum Tolerated Dose of Irinotecan Based on *UGT1A1* Genotypes: A Systematic Review

**DOI:** 10.3390/pharmaceutics17050542

**Published:** 2025-04-22

**Authors:** Xando Díaz-Villamarín, María Teresa Nieto-Sánchez, María Martínez-Pérez, Paula Novo-González, Emilio Fernández-Varón, Alicia Torres-García, Beatriz González Astorga, Isabel Blancas, José Cabeza-Barrera, Rocío Morón

**Affiliations:** 1 Instituto de Investigación Biosanitaria de Granada (Ibs. Granada), 18012 Granada, Spain; 2 Hospital Pharmacy, Hospital Universitario San Cecilio, 18016 Granada, Spain; 3 Hospital Pharmacy, Hospital Regional Severo Ochoa, 28914 Leganés, Spain; 4 Department of Pharmacology, Center for Biomedical Research (CIBM), University of Granada, 18016 Granada, Spain; 5 Medical Oncology, Hospital Universitario San Cecilio, 18016 Granada, Spain

**Keywords:** irinotecan, *UGT1A1*, maximum tolerated dose, pharmacogenetics, pharmacogenomic, personalized medicine, clinical pharmacy

## Abstract

**Background/Objectives:** Irinotecan is used in monotherapy or combined with other drugs for treating different cancer streams. SN-38, the active metabolite of irinotecan, is 70% inactivated by the uridine diphosphate (UDP) glucuronosyltransferase family 1 member A1 (UGT1A1) enzyme. The UGT1A1*6 (rs4148323) and *28 (rs3064744) alleles in the gene encoding the enzyme lead to decreased enzyme expression and increased severe irinotecan toxicity. Carrying one or two copies of these alleles results in a UGT1A1 intermediate or poor metabolizer status (IM, PM). The Food and Drug Administration (FDA)-approved drug labels and European Medicines Agency (EMA) European Public Assessment Report (EPAR) for irinotecan recommend dose adjustments based on *UGT1A1* genotypes, but only for UGT1A1 PM patients. However, available pharmacogenetic (PGx) dosing guidelines for the *UGT1A1*–irinotecan interaction lack a consensus about considered genetic variants, genotype-translated phenotypes, and therapeutic recommendations. We aimed to describe evidence regarding the impact of the *UGT1A1* genotype in irinotecan toxicity to inform irinotecan-dosing recommendations based on possible *UGT1A1* genotypes. **Methods**: A systematic review was performed to find all the Phase I clinical trials looking for the maximum tolerated dose (MTD) or dose-limiting toxicities (DLTs) of irinotecan depending on the *UGT1A1* genotype. **Results**: Toxicity-related events and the MTD of irinotecan differ among UGT1A1 normal metabolizers (NM), IM, and PM patients considering the UGT1A1*28 and/or *6 variants. **Conclusions**: Dose adjustments might also be recommended for UGT1A1 IM patients (*1/*28 or *1/*6 genotypes), with a 15% dose reduction considered.

## 1. Introduction

Irinotecan is a camptothecin analogue developed in the 1990s to address its solubility issues. Camptothecin, a cytotoxic agent from *Camptotheca acuminata*, was developed as an anticancer agent in the early 1970s [1].

Irinotecan (also known as CPT-11) was first approved by the Food and Drug Administration (FDA) in 1996 [2] and remains a cornerstone drug in the treatment of colorectal, pancreatic, and gastroesophageal cancer. It may be found as hydrochloride or as liposome injections.

Irinotecan is used in monotherapy or combined with other drugs in multiple chemotherapy schemes, mainly with leucovorin, 5-fluorouracil (5-FU), and oxaliplatin (FOLFIRINOX) in the adjuvant treatment of pancreatic cancer and also as a first-line treatment for metastatic colorectal cancer (mCRC) in combination with 5-FU and leucovorin (FOLFIRI) or with cisplatin in other neoplasms, such as lung and ovarian cancer [3].

Irinotecan is a prodrug that is metabolized in the liver and intestine through hydrolysis by carboxylesterases (CES1 and CES2) to activate the active component, SN-38 [1]. This active metabolite is an inhibitor of DNA topoisomerase I that causes the death of cancer cells [2]. SN-38 is cytotoxic and destabilizes the covalent topoisomerase I–DNA complex formed in cancer cells, causing irreversible double-strand breaks that lead to S-phase arrest followed by cell death [2]. Much of the antitumor activity of irinotecan is derived from its fast conversion to the active metabolite SN-38. This metabolite contributes to dose-limiting toxicities (DLTs), such as myelotoxicity, neutropenia, and diarrhea [4,5].

**Figure 1:**
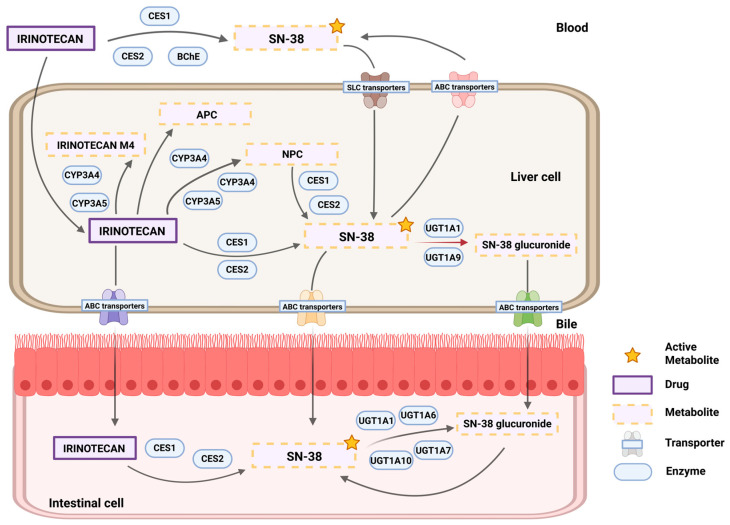
Irinotecan pathway. Abbreviations: CES1-2, carboxylesterase type 1 and 2; BchE, butyrylcholinesterase; SN-38, active metabolite of irinotecan; SLC, solute carrier; ABC transporters, ATP-binding cassette transporter; APC, 7-ethyl-10-[4-N-(5-aminopentanoic acid)-1-piperidino]carbonyloxycamptothecin; NPC, 7-ethyl-10-[4-(1-piperidino)-1-amino]carbonyloxycamptothecin; CYP3A4-3A5, cytochrome P450 3A4 and 3A5 isoenzymes; UGT1A1, uridindiphosphate (UDP) glucuronosyltransferase family 1 member A1 *. * 1 to 10 means other UDP-glucuronosyltransferase isoenzymes of the UGT1A sub-family.

Approximately 70% of SN-38 is inactivated in the liver through its conjugation with glucuronic acid, a reaction mediated by the uridine diphosphate (UDP) glucuronosyltransferase (UGT) family 1 member A1 (UGT1A1) enzyme [6]. The resulting metabolite is SN-38G, practically inactive, which will go to the intestine for elimination [6]. In the intestinal lumen, bacterial beta-glucuronidases can reverse the reaction and transform the inactive SN-38G back into the active form of SN-38 [7,8]. This is a factor that contributes to toxicity, specifically to the appearance of late dose-limiting diarrhea. The remaining 30% of irinotecan is inactivated through oxidation to the inactive metabolites APC (7-ethyl-10-[4-N-(5-aminopentanoic acid)-1-piperidino]carbonyloxycamptothecin) and NPC (7-ethyl-10-[4-(1-piperidino)-1-amino]carbonyloxycamptothecin). The NPC can be further metabolized to SN-38 by CES1 and CES2 (Figure 1) [9,10].

Irinotecan metabolism results in three major DLTs: myelosuppression, diarrhea, and neutropenia. All three may vary in response to *UGT1A1* gene variation, as UGT1A1 mediates the detoxification and conjugation of the active component SN-38 [4].

Neutropenia is directly related to the plasma concentration of SN-38. Late-onset diarrhea is caused by the excessive biliary secretion of SN-38 into the lumen of the intestine. An estimated 70% of patients receiving irinotecan therapy experience diarrhea, making proper dosing difficult [7]. Approximately 55% of these patients receive an underdose after one month. Acute diarrhea is caused by the anticholinesterase activity of irinotecan [11,12], which destroys the secretory and absorptive functions and properties of the intestinal mucosa. SN-38 has mitotic-inhibitory properties, which also contributes to structural and functional defects in the intestinal lumen [6].

If the glucuronidation rate of SN-38 in the intestines is low, active SN-38 is exposed to epithelial cells for a longer period, leading to severe diarrhea. Therefore, the glucuronidation of SN-38 is necessary to limit gastrointestinal (GI) toxicity. Once SN-38G is excreted in the feces, there is a risk that it may be reactivated to SN-38 by bacterial beta-glucuronidases. Active SN-38 further destroys intestinal epithelial cells, leading to delayed severe diarrhea [9,13,14].

### 1.1. UGT1A1: Uridine Diphosphate (UDP) Glucuronosyltransferase Family 1 Member A1

The *UGT1A1* gene encodes a UDP-glucuronosyltransferase, an enzyme participating in the glucuronidation pathway transforming small lipophilic molecules, as SN-38, into water-soluble, excretable metabolites. *UGT1A1* is part of a complex locus encoding several UDP-glucuronosyltransferases. This locus includes thirteen unique alternate first exons (four pseudogenes among them) and four common exons. Each of the remaining first exons encodes the substrate-binding site, is regulated by its own promoter, and may be spliced to the common exons, resulting in nine proteins with different N-termini and identical C-termini. The main substrate of the UGT1A1 enzyme is bilirubin, although it also has moderate activity with simple phenols, flavones, and C18 [15,16].

Many genetic variants are known for *UGT1A1* (Table 1). Among them, UGT1A1*28 and *6 have been associated with a variable response to irinotecan with the highest level of evidence [17].

According to the Human Genome Variation Society (HGVS) Nomenclature [20], the UGT1A1*28 and *37 alleles (rs8175347) are characterized by having a seventh and eighth dinucleotide repeat in the TATA box of the promoter region, respectively (TA8 or TA9 in total), unlike the UGT1A1*1 allele with six repeats (TA7 in total) [17]. UGT1A1*28 and *37 may be found in the literature as A(TA)7TAA and A(TA)8TAA; to avoid confusion, in advance, we will always refer the *allele. Anyway, the higher number of repeats results in lower rates of transcription and enzymatic activity [21,22]. With a decrease in these rates, there is a decrease in the detoxification of SN-38 and the exposure time of active SN-38 in the intestines is prolonged. Therefore, patients homozygous or heterozygous for the UGT1A1*28 or *37 alleles commonly develop severe dose-limiting neutropenia and late diarrhea [4,5,23]. UGT1A1*6 (G > A, rs4148323) leads to increased toxicity as occurs with the UGT1A1*28 and *37 alleles [24,25,26,27]. UGT1A1*6 may lead to decreased enzymatic activity in either the heterozygous or homozygous form. Patients homozygous for the UGT1A1*6 allele (UGT1A1*6/*6 genotype) are at a high risk for grade 4 (G4) neutropenia [4,9,25]. Individuals heterozygous for the UGT1A1*6 or *28 alleles (UGT1A1*1/*6 and *1/*28 genotypes) often undergo higher rates of toxicity, as these alleles can have up to half the activity of the glucuronidating enzyme compared to UGT1A1*1 [14]. The other alleles with an impact on UGT1A1 enzyme function are UGT1A1*36, characterized by a sixth dinucleotide repeat in the TATA box, *27, and *80. UGT1A1*27 and *36 have been related to a variable response to different drugs but not to irinotecan [17].

According to information from Pharmgkb [17], the UGT1A1*80 allele is in very high linkage disequilibrium with *28 and *37, which is reflected by the *80 + *28 and *80 + *37 alleles entries (Table 1). Currently, the function of the UGT1A1*80 allele alone, with the absence of *28 or *37, is uncertain.

The differences in the distribution of *UGT1A1* variants in different populations are interesting and may be relevant from a clinical perspective. UGT1A1*28 is prevalent in Caucasian and African American populations with frequencies of 39% and 43%, respectively [22,28], with a lower frequency in the East Asian population (approx. 16%). In contrast, the UGT1A1*6 variant (rs4148323) has an approximate frequency of 16% in Asians according to the Allele Frequency Aggregator (ALFA) [19] and the 1000 Genomes Project [18]. In the 1000 Genomes, the Asian population represents *n* = 1000 of *n* = 5008 of total genotyped individuals, which explains the difference between the Iberian Peninsula (IBS) and global allele frequencies (0% vs. 3.43%, respectively). In contrast, ALFA reports a total frequency of 0.7% because Asians are only *n* = 7018 out of *n* = 338,730 in total.

Regarding *UGT1A1* rs3064744, which characterizes the *28 and *37 alleles, according to data from the Genomes Aggregation Database (gnomAD) (Genomes Aggregation Database) [29] retrieved from the Ensembl [30], 61.71% of patients carry TA7, 34.61% TA8, 2.21% TA6, and 1.47% TA9.

### 1.2. Pharmacogenetics of the UGT1A1–Irinotecan Interaction

The irinotecan active metabolite SN-38 is further metabolized via UGT1A1. Genetic variants of the *UGT1A1* gene, such as the UGT1A1*28 (TA8), *37 (TA9) and *6 alleles, lead to reduced UGT1A1 enzyme expression or activity and decreased function to a similar extent [27].

Individuals who are heterozygous for either the UGT1A1*28 or *6 alleles (*1/*28, *1/*6) are intermediate metabolizers (IMs). Individuals who are homozygous or compound (double) heterozygous for these alleles (UGT1A1*28/*28, *6/*6, and *6/*28 genotypes) are UGT1A1 poor metabolizers (PMs) [17].

The FDA-approved drug label for irinotecan hydrochloride (Camptosar^®^, Pfizer, NY, USA) [17] states that UGT1A1 PM patients experience increased systemic exposure to SN-38 (active metabolite of irinotecan), being at an increased risk for severe or life-threatening neutropenia and diarrhea during treatment. It also states that UGT1A1 IM individuals may also have an increased risk of severe or life-threatening neutropenia and diarrhea. It also recommends *UGT1A1* genotype testing for the *28 and *6 alleles to determine the UGT1A1 metabolizing status, considering a reduction in the starting dose of irinotecan by at least one level for homozygous or compound heterozygous patients regarding the UGT1A1*28 and/or *6 alleles (UGT1A1*28/*28, *6/*6, or *6/*28 genotypes), closely monitoring patients with UGT1A1*28 or *6 alleles for neutropenia during and after treatment, and subsequent dosage modifications based on individual patient tolerance to treatment.

Also, the FDA-approved drug label [31] and the European Medicines Agency (EMA) European Public Assessment Report (EPAR) [32] for irinotecan liposome injection (Onyvide^®^) recommend a starting dose of 50 mg/m^2^ in patients known to be homozygous for the UGT1A1*28 allele and to increase the dose to 70 mg/m^2^ as tolerated in subsequent cycles.

The Dutch Pharmacogenetics Working Group (DPWG) from the Royal Dutch Pharmacists Association considers *UGT1A1* genotyping to be essential and that it must be performed before treatments with irinotecan are started [27]. In their dosing guidelines based on pharmacogenetic (PGx) information, the DPWG recommends, for UGT1A1 PM patients, to start with 70% of the normal dose, considering that the dose can be increased if the patient tolerates this initial dose, guided by the neutrophil count. On the other hand, in UGT1A1 IM patients, the DPWG guidelines report that no action is needed for this gene–drug interaction [27]. These guidelines, provided as Supplementary Materials, include a list of *UGT1A1* alleles and the corresponding metabolic capacity, to translate *UGT1A1* phenotypes.

In Spain, the recent update of the services portfolio of the National Health System (*Sistema Nacional de Salud*, SNS) included the irinotecan–*UGT1A1* interaction among the PGx test to be offered by the public health system [33] and the Spanish Society of Pharmacogenetics and Pharmacogenomics (*Sociedad Española de Farmacogenética y Farmacogenómica*, SEFF) developed dosing guidelines considering this drug–gene interaction [34]. These PGx dosing guidelines do not provide dosing recommendations for patients carrying none or one *UGT1A1* mutated allele, in accordance with DPWG guidelines. On the other hand, for patients carrying two mutated alleles among UGT1A1*6, *28, and *37 alleles, these guidelines recommend starting the irinotecan treatment with 70–75% of the standard dose if the needed irinotecan dose is ≥180 mg/m^2^.

The European Society for Medical Oncology (ESMO) suggests the *UGT1A1* genotyping is an option in patients with suspected decreased UGT1A1 function or a planned dose higher than >180 mg/m^2^ [35], and the Clinical Pharmacogenenetic Implementation Consortium (CPIC) reports the Irinotecan–*UGT1A1* interaction with the level of evidence of 1A, thereby relevant enough to use the PGx information to individualize the pharmacotherapy [36].

Also, the main authorities in this regard in Canada (Canadian Pharmacogenomics Network for Drug Safety, CPNDS), France (French National Network of Pharmacogenetics, RNPGx) [37], or Italy (*Gruppo di Lavoro di Associazione Italiana di Oncologia Medica—Società Italiana di Farmacologia*) [38] recommend *UGT1A1* genotyping before the start of irinotecan and/or dose reductions in UGT1A1 PM patients.

As we can see, there is surplus evidence supporting the importance of *UGT1A1* genotyping in intended-to-treat patients with irinotecan. On the other hand, there is no consensus about which *UGT1A1* variants should be tested or the *UGT1A1* phenotype-translated dosing recommendations, and it is difficult to find the criteria used for considering information to be relevant.

With this systematic review, we aimed to describe the evidence regarding the impact of the *UGT1A1* genotype on irinotecan levels and translated adverse drug events (ADEs). This might help in the decision to provide a PGx-based therapeutic recommendation depending on possible *UGT1A1* genotypes for irinotecan-treated patients.

## 2. Materials and Methods

### 2.1. Search Strategy and Inclusion/Exclusion Criteria

A systematic review in compliance with the PRISMA guidelines [39] was performed to find all Phase I clinical trials looking for the maximum tolerated dose (MTD) or DLTs of irinotecan depending on the *UGT1A1* genotype and reporting peer reviewed results in relevant journals. We searched Pubmed on 2 April 2024 for randomized controlled trials or clinical trials using the following argument: (Irinotecan or Folfirinox or Folfiri) and (pharmacogenetics or pharmacogenomics or *UGT1A1*), without considering a date range or language filters.

We also manually checked the bibliography provided in PharmGKB [17] to ensure that all the relevant manuscripts about this topic were included.

The articles found in the initial search were assessed regarding the following inclusion/exclusion criteria. We excluded articles not written in English, not published in scientific journal indexed in the Journal Citation Report (JCR), not reporting results in humans, or not proving results about the DLT or MTD of irinotecan, or any antineoplastic scheme including this drug, depending on the *UGT1A1* genotype. Therefore, we included research articles reporting results about Phase I trials, including patients receiving irinotecan based on *UGT1A1* genotypes only.

In this regard, we first checked the records (titles and abstracts) found in the initial search to exclude those not written in English or not providing results about patients (e.g., study protocols), then we carefully checked the methodology to exclude those articles not testing variants of the *UGT1A1* gene among patients, and we finally categorized the remaining publications into four groups. The first group included studies not using the *UGT1A1* genotype for dose tailoring (e.g., as an inclusion/exclusion criterion of patients in the trial, as a covariate, etc.), and the second group covered research articles reporting results about clinical trials comparing an intervention group receiving a PGx-dose-tailored treatment and a control group not PGx-tested, both receiving irinotecan. The third category included articles reporting the results of Phase II or III clinical trials examining the efficacy of irinotecan. The fourth group included those Phase I clinical trials examining the DLT and/or MTD of irinotecan depending on the *UGT1A1* genotype. Research articles in group 4 were finally included.

Other Information: The review was not registered, and a specific protocol was not prepared.

### 2.2. Data Extraction and Quality Assessment

The search strategy was performed by five researchers. First, the publications found in the original search were cross-over assessed for meeting the inclusion/exclusion criteria and categorized into the groups described above by four of those researchers. The discrepancies were assessed by all four plus one other researcher, and articles were finally included when three among them agreed with the inclusion/exclusion decision.

A quality assessment of the publications meeting the inclusion criteria was performed using the Newcastle–Ottawa quality assessment Scale (NOS) [40]. Each study was assessed based on three categories (selection, comparability, and exposure) and eight items, up to nine “stars/points”, as the top score. Articles with an NOS score under five points were finally excluded.

We also appraised the studies to be included in systematic reviews using the Grading of Recommendations, Assessment, Development, and Evaluations (GRADE) criteria [41].

The following information from the included studies was recorded: author, cancer type (study population), anticancer scheme containing irinotecan, ethnicity, follow-up time, number of patients, studied *UGT1A1* variants, reference single-nucleotide polymorphism (SNP) (rs), studied endpoint, minor allele frequency (MAF), and genotypes of recruited patients. Among the Phase I trials found, we collected the reported results in each publication about DLT and MTD depending on the patients’ *UGT1A1* genotype and administered irinotecan dose, including the number of patients for each *UGT1A1* genotype and/or the irinotecan dose level, the number of patients showing a DLT in each dose level, and the calculated MTD in each study based on these data. Also, we collected data about reported ADEs during follow-up in each study, categorizing these ADEs as hematological (neutropenia, leukopenia, or others) and non-hematological (nausea or vomiting, diarrhea, or others). We just reported ADEs during the first or second chemotherapy cycle including irinotecan since these were the data used to assess the DLTs and calculate MTDs.

Furthermore, we collected data about the reported results on pharmacokinetic (PK) analyses when the results were categorized depending on the *UGT1A1* genotype. We provide results of the area under the curve (AUC) of SN38 and SN38G and the AUC ratio between both parameters. We self-calculated the AUC ratio when this was not provided.

Patients from each study were categorized, regardless of the tested *UGT1A1* variants, as wild-type if they did not carry any mutated allele and as heterozygous or recessive homozygous when carrying one or two mutated alleles, respectively. This means that for publications including patients tested for the UGT1A1*28 and *6 alleles, the heterozygous group includes patients with the UGT1A1*1/*28 or UGT1A1*1/*6 genotypes.

## 3. Results

In the initial search, we found *n* = 158 publications. First, we excluded *n* = 1 publication not written in English, *n* = 2 study protocols, *n* = 1 reporting results about the usefulness of different lab procedures, and *n* = 25 articles testing genetic variants not in *UGT1A1*. We also excluded *n* = 1 article that was not available.

**Figure 2:**
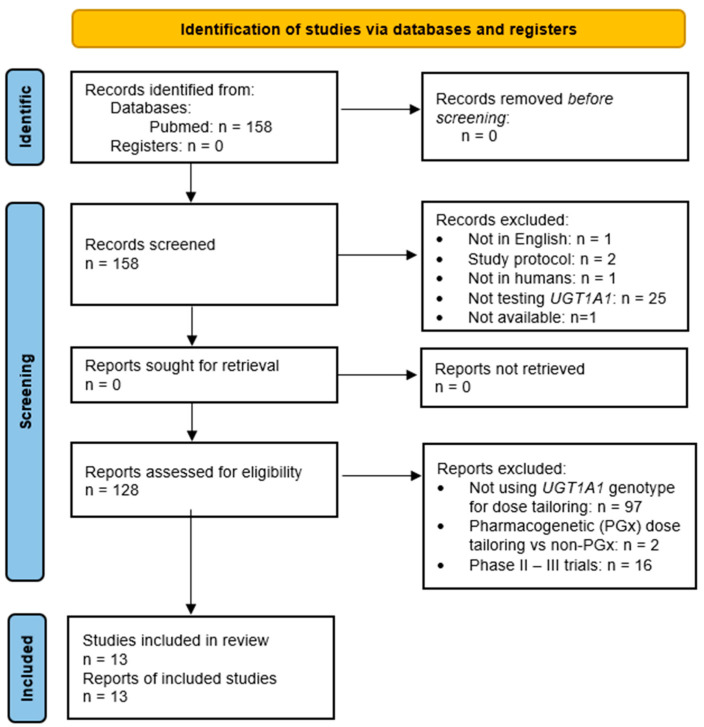
Search strategy (modified from PRISMA 2020 flow diagram) [39].

Among the remaining publications (*n* = 128), *n* = 97 were categorized in the first group, *n* = 2 in the second one, and *n* = 16 in the third group, thus excluded (Figure 2). This means that we finally found *n* = 13 research articles reporting results about Phase I clinical trials reporting DLTs and calculating the MTDs of irinotecan based on *UGT1A1* genotypes.

Among the included publications, none were scored below five points with the NOS scale, and we did not find evidence of publication bias after Harbord’s test. According to the GRADE criteria, we categorized as the evidence about the differences in MTDs and DLTs depending on *UGT1A1* genotypes as high GRADE considering the UGT1A1*6 and/or 28 alleles and as high–moderate if we considered the differences among carrying none, one, or two mutated alleles.

We found *n* = 13 Phase I clinical trials examining the *UGT1A1*–irinotecan interaction (Table 2) and one among them also reporting Phase II results [42] comparing the efficacy of FOLFIRINOX vs. FOLFIRABRAX (5-fluorouracil, leucovorin, irinotecan, and nab-paclitaxel) schemes. All of them considered the UGT1A1*28 allele, and only four also tested the UGT1A1*6 for irinotecan dose tailoring; five out of twelve studies were performed on the Asian population (total, *n* = 237 patients) and *n* = 7 on populations with a majority of white patients (>80% of total *n* = 354), all of them considering the first cycle of the treatments to assess DLTs and calculate the MTD, except the study by Satoh T. et al. [43], considering two cycles.

Among the included articles, two trials by Satoh T. et al. [43] and Innocenti F. et al. [44] aimed to determine the MTD of irinotecan used in monotherapy but with many differences. Satoh T. et al. recruited patients with GI cancer whose *UGT1A1* genotypes were UGT1A1*28/*28, *6/*6, or *28/*6, while Innocenti F. et al. included patients with different diagnoses and just considered the UGT1A1*28 variant. Anyway, both studies found an influence of the *UGT1A1* genotype on the irinotecan MTD, safety, and PK. Satoh T. et al. [43] found that starting at high doses (>150 mg/m^2^) would be very risky in patients carrying two UGT1A1*28 and/or *6 alleles, so, although they can receive irinotecan at a starting dose of 150 mg/m^2^, they must be closely observed at least during the first cycle. Innocenti F. et al. [44] concluded that the tolerable doses of irinotecan range from 400 to 850 mg based on the UGT1A1*28 genotype.

We also found Phase-I clinical trials not only assessing irinotecan-treated patients in monotherapy regimens. Goetz M.P. et al. [42] aimed to define the MTD of the CAPIRINOX (capecitabine, irinotecan, and oxaliplatin) regimen depending on the *UGT1A1* genotype in different cancer streams, but just considering the UGT1A1*28 variant, and concluding that the UGT1A1*28 genotype affects the tolerable dose and PK of the CAPIRINOX regimen. Also, Joshi S.S. et al. [45] assessed the safety and tolerability of FOLFIRABRAX in locally advanced or metastatic GI cancer patients considering the *UGT1A1* genotype-guided dosing of irinotecan. They also confirmed that a genotype-dosing based on the UGT1A1*28 genotype is tolerable. Ng M. et al. [46] performed a Phase 1b open-label, prospective study to assess the safety and tolerability of the OXIRI (oxaliplatin, chronomodulated capecitabine, and irinotecan) regimen for pancreatic adenocarcinoma patients. Regarding the irinotecan–*UGT1A1* interaction, they found that the dose tailoring of irinotecan in accordance with the patient’s *UGT1A1* genotype may further improve the safety of the OXIRI regimen.

Studies by Kim K. P. et al. [47] and Zhu J. et al. [48] both examined the safety and efficacy of XELIRI (capecitabine and irinotecan; also known as CAPIRI) based on the *UGT1A1* genotype. The first one reported the feasibility of the irinotecan dose tailoring based on the UGT1A1*28 and *6 genotype in Korean patients with mCRC in a XELIRI scheme. They also reported that the administration of higher doses of irinotecan in patients carrying two *UGT1A1* mutated alleles is safe.

Three other trials studied this among mCRC patients treated with FOLFIRI [49,50,51]. Toffoli G. et al. [49] aimed to identify the MTD among UGT1A1*1/*1 and *1/*28 patients treated with FOLFIRI plus bevacizumab and to assess the influence of bevacizumab on the irinotecan PK. They observed that, among these patients, higher-than-standard doses of irinotecan can be safely given to patients with UGT1A1*1/*1 and *1/*28 genotypes and highlighted that most patients may be underdosed with respect to irinotecan.

In one other study by Toffoli G. et al. [51], they looked for the MTD of irinotecan in patients with mCRC with UGT1A1*1/*1 and *1/*28 genotypes and found that 180 mg/m^2^ of irinotecan in a FOLFIRI regimen is considerably lower than the tolerated dose when patients with the UGT1A1*28/*28 genotype are excluded. The third among studies treating patients with a FOLFIRI regimen, by Kim K. P. et al. [50], found that escalated doses of irinotecan plus fixed doses of simplified 5-FU and leucovorin (sLV5FU2) were feasible for Korean mCRC patients carrying a UGT1A1*1/*28, *1/*6, or wild-type genotypes.

Among the Phase I trials found (Table 2), there were three studies [52,53,54] assessing the safety of novel preparations of irinotecan, the IHL-305, a novel pegylated liposome containing irinotecan [52]; the NK012, a macromolecular polymeric micelle formulation of SN-38 [53]; and the nal-IRI, an irinotecan liposome injection. These were not specifically focused on determining differences depending on the *UGT1A1* genotype. Anyway, they categorized patients depending on the *UGT1A1* profile and found different MTDs based on this, as it is described below (Table 3).

### 3.1. Dose-Limiting Toxicities and Maximum-Tolerated Doses

Studies shown in Table 2 consider quite similar ADEs as DLTs (Table 3), all of them according to the National Cancer Institute (NCI) Common Terminology Criteria for Adverse Events (version 3 or 4). Grade 3 or 4 (G3-4) hematological ADEs, including neutropenia, febrile neutropenia, or thrombocytopenia, were especially considered and the most common ADEs reported. Included studies differ among non-hematological ADEs considered DLTs although most of them included G3-4 diarrhea, nausea or vomiting, and irinotecan treatment delays. They also show different dose-escalation schemes and characteristics to calculate the MTD. Most of them consider the MTD when between 25% to 33% of patients showed at least one DLT during the first cycle of treatments.

Anyway, all trials examining DLTs and MTDs depending on the *UGT1A1* genotype found significant differences, but two studies did not provide results depending on the *UGT1A1* genotype [52,53], and one did not suggest MTDs [45].

Five studies [44,48,49,50,51] found higher rates of DLTs resulting in a higher MTD in *UGT1A1*-heterozygous patients considering the UGT1A1*28 and/or *6 alleles (UGT1A1*1/*6 or *1/*28 genotypes), compared to the *UGT1A1* wild-type genotype (UGT1A1*1/*1). In this regard, differences in the rates of DLTs and MTDs were even higher between *UGT1A1*-homozygous genotype patients (UGT1A1*28/*28, *6/*6, and *6/*28) compared to *UGT1A1*-wild-type or heterozygous.

On the other hand, results in four studies suggest the same MTD for wild-type and heterozygous patients but lower for homozygous [42,43,46,47,48]. Among them, one study did not provide results about DLTs and irinotecan doses [46]; one study [47] found differences in DLT rates at the same irinotecan dose level among wild-type, heterozygous, and homozygous patients but, according to its methodology, resulted in the same MTD for wild-types and heterozygous; and one more other study [42] suggests the same irinotecan doses but combined with different capecitabine doses for each *UGT1A1* genotype.

The ADEs reported by *UGT1A1* genotype reported in each study are shown in Supplementary Table S1.

### 3.2. Pharmacokinetics

Among the research articles providing PK test results (“endpoint” column in Table 2), no information was extracted from four articles [49,51,52,53], because results by *UGT1A1* genotype were not provided or because these results could not be calculated from the information contained in each of the publications.

Furthermore, Clarke J. L. et al. [54] did not provide AUC data for SN-38 or SN-38G, although they did provide maximum concentration (Cmax) data per dose, and considering the scaling described in the methodology, Cmax results can be extrapolated based on *UGT1A1* genotype.

Finally, PK information of irinotecan depending on the *UGT1A1* genotype could be obtained from six studies (Table 4). As we can see, the AUC ratio (AUC SN38G/AUC SN38) in most of the studies is different depending on the *UGT1A1* genotype, even if we compare the wild-type genotype with carrying just one mutated allele.

## 4. Discussion

UGT1A1 is a key enzyme in the detoxification of irinotecan. Different genetic variants have been identified in the gene encoding the enzyme that have been shown to reduce the activity of the enzyme. Thus, individuals carrying these variants could reach a toxic concentration of the active metabolite when treated with normal doses. On the other hand, there is a risk of underdosing patients carrying these *UGT1A1* variants when trying to avoid the appearance of serious ADEs. This makes it essential to conduct clinical trials considering the *UGT1A1* genotype for the calculation of MTDs.

The irinotecan–*UGT1A1* interaction is relatively well known and has led to the drug’s summary of product characteristics (SmPC) taking this interaction into account for drug dosing and to the existence of irinotecan dosing guidelines based on PGx information.

The PGx guidelines for the irinotecan–*UGT1A1* interaction agree on not recommending dose adjustments for patients carrying a single mutated allele and starting with 70% of the usual dose for patients carrying two mutated *UGT1A1* alleles, with special consideration to UGT1A1*28.

In this regard, there is no consensus regarding the variants in *UGT1A1* that have demonstrated their influence on the toxicity or efficacy of irinotecan. In addition, irinotecan or its active metabolite is administered in different pharmaceutical forms, in monotherapy or combined with other drugs in different therapeutic regimens, at different doses regardless of the patient’s *UGT1A1* genotype, and in the treatment of different cancer streams. Thus, it could be the case that all of these variables could be influencing the association of the *UGT1A1* genotype with the response of patients to irinotecan.

With this study, we try to provide information in this regard, summarizing the results and conclusions of all Phase I clinical trials published in high-impact journals that have assessed the influence of the *UGT1A1* genotype on the calculation of the MTD of irinotecan in different therapeutic regimens or for different pharmaceutical forms.

### 4.1. Limitations

We limited the search to PubMed and filtered for “clinical trial” or “randomized clinical trials”, so some trials might have been missed in the search. Furthermore, we did not consider articles in Embase, Cochrane, Scopus, and grey literature, such as conference papers or clinical trial registries, so publication bias is possible. On the other hand, this search strategy was considered to only consider publications with the results of trials with a sufficient scientific level, thereby published in high-impact journals indexed in the JCR and peer reviewed. Therefore, candidate publications to be included have also been evaluated with the NOS scale. Also, in this regard, maybe a different method might be used to appraise the included studies. We considered using the NOS scale since these are not Phase II-III trials assessing treatments or intervention but Phase I trials looking for the MTD that also categorizes patients and finds differences depending on *UGT1A1* genotypes.

To statistically confirm the results, a meta-analysis might be carried out. On the other hand, we considered that it had no sense regarding the variability among included trials, including different populations, treatment schemes, endpoints, etc.

We did not consider efficacy, although these regimens have already demonstrated efficacy in Phase II and III trials, and patients who suffer from a DLT in real clinical practice are withdrawn from treatment. This is not the aim of the study but rather to provide a guide for dose adjustments based on the *UGT1A1* genotype for each regimen to avoid DLTs in the first cycle of treatment.

### 4.2. Safety of Irinotecan-Containing Schemes

It is important to consider the relationship between the risk of overdose or underdose of irinotecan in original trials that did not consider the genotype. Irinotecan is administered based on the patient’s surface area (mg/m^2^) and has a high associated rate of ADEs, especially hematological events (mainly neutropenia/leucopenia) and other non-hematological events, such as diarrhea and asthenia or fatigue. For this reason, it is important to adjust the dose administered to the patient by as much as possible, avoiding the high risk of ADEs but also the risk of underdosing the drug and lack of treatment efficacy.

Initially, the recommended dose of irinotecan was 180 or 350 mg/m^2^, combined or in monotherapy, respectively, without considering the influence of the UGT1A1 metabolizing status. This grouped patients who, based on their *UGT1A1* genotype and metabolizer status, could have received a lower dose of the drug to avoid ADEs or a higher dose to improve therapy without increasing the risk of ADEs.

The clinical trials found in this systematic review confirm the safety profile of the drug (Supplementary Table S1). Even, Innocenti F. et al. [44] comment that the toxicity profile of individualized irinotecan dosing does not change, highlighting that neutropenia is the dominant DLT, and diarrhea contributes to a lesser extent, and also that those ADEs are likely pharmacodynamic consequences of higher exposure to SN-38, which is in part determined by the UGT1A1*28 variant [55,56,57,58].

### 4.3. Usefulness of UGT1A1 Genotyping for Cancer Treatment Using Irinotecan

This systematic review confirms the usefulness of UGT1A1*28 and/or *6 genotyping prior to the treatment start with irinotecan, regardless of the chemotherapy regimen and the type of cancer, leading to different MTDs depending on the genotype and confirming these results with PK studies in some of the included trials (Table 4).

On the other hand, these trials also demonstrate that there are other variables influencing the appearance of DLTs and, therefore, the calculation of MTDs, beyond the patient’s genetic profile. Thus, the time between chemotherapy cycles [52], the chemotherapy regimen, and especially the dose of capecitabine combined with irinotecan [42] seem to be determining factors in the appearance of DLTs.

Also, the variability observed between the clinical trials found could be explained by factors that were not considered, such as other genetic variants in *UGT1A1* or other genes related to the drug pathway, to the degree of gene expression (epigenetics), or clinical parameters, such as the ancestry of the recruited patients. Even so, this variability might be explained by the interaction of many of these characteristics. In this regard, we know that the distribution of *UGT1A1* variants differs by population, and relevant variants had not been tested in the included trials.

In any case, the studies found in this review highlight that it would be better to carry out the PGx test before the treatment starts and after a full explanation to all patients [43] and that the *UGT1A1* genotype is an important determinant of the irinotecan drug response and provides further evidence to support the use of *UGT1A1* genotyping to determine the dose of irinotecan-based chemotherapy [42], suggesting that the optimal dosage of irinotecan-containing agents can be evaluated based on the *UGT1A1* genotype information to maximize their efficacy and minimize toxicity [47], and also that the *UGT1A1* genotype is a parameter affecting the irinotecan PKs and a factor for judging the risk of hemotoxicity [43], but also that further studies are necessary to assess whether higher doses of irinotecan can be stratified by *UGT1A1* genotypes [50].

We can see that the usefulness of *UGT1A1* genotyping prior to the start of treatment with irinotecan is confirmed, although it is also true that there is a risk of underdosing in patients whose dose is reduced based on their genotype, as highlighted by Toffoli G. et al. [49]. A dose reduction to avoid the accumulation of SN-38, due to its reduced conversion to SN-38G, may result in not reaching the minimum effective concentrations. In this sense, monitoring SN-38 levels during the first cycles of treatment with chemotherapy regimens that include irinotecan could be useful.

In summary, the PGx *UGT1A1* test is useful to avoid serious early ADEs, and PK tests could be useful to ensure the efficacy of the treatment and prevent late ADEs.

### 4.4. Insights Related to Pharmacogenetic Dosing Guidelines and Irinotecan SmPC

Irinotecan is used in different types of cancer combined in different chemotherapy regimens. Anyway, the FDA-approved SmPC for irinotecan hydrochloride (CAMPTOSAR^®^) recommends the consideration of a reduction in the starting dose by at least one level for patients known to be homozygous for the UGT1A1*28 or *6 alleles (UGT1A1*28/*28 or *6/*6 genotypes) or compound heterozygous for the UGT1A1*28 and *6 alleles (UGT1A1*6/*28), comments that individuals who are heterozygous for either the UGT1A1*28 or *6 alleles (UGT1A1*1/*6 or *1/*28) are UGT1A1 IM and may also have an increased risk of severe or life-threatening neutropenia, and that the published studies have shown that individuals with UGT1A1*28 and *6 alleles may be at an increased risk of severe diarrhea. In addition, it recommends the close monitoring of patients with UGT1A1*28 or *6 alleles for neutropenia during and after treatment with CAMPTOSAR^®^.

On the other hand, the drug label for ONIVYDE^®^ (Ipsen Biopharm Ltd., MA, USA), a liposomal form of irinotecan, recommends a reduced starting dose of ONIVYDE of 50 mg/m^2^ for patients known to be homozygous for the UGT1A1*28 allele and a dose increase of ONIVYDE to 70 mg/m^2^ if tolerated, in subsequent cycles.

However, the CPIC has not developed guidelines for the irinotecan–*UGT1A1* interaction, and the DPWG guidelines recommend, in patients carrying two mutated alleles, considering the variants indicated in Table 1, to start with 70% of the normal dose if the patient tolerates this initial dose, commenting that the dose can be increased, guided by the neutrophil count, and not to take any measures in patients carrying only one *UGT1A1*-mutated allele.

As we can see in the results, in the Phase I trials, different MTDs have been found depending on the *UGT1A1* genotype in patients treated with irinotecan in different regimens and for different pharmaceutical forms.

As we detail, five studies [44,48,49,50,51] found higher rates of DLTs resulting in a lower MTD in *UGT1A1*-heterozygous patients considering the UGT1A1*28 and/or *6 alleles compared to the *UGT1A1*-wild-type genotype, but four studies suggest the same MTD for wild-type and heterozygous patients but lower for homozygous [42,43,46,47]. But, among those four studies, one does not provide results about DLTs and irinotecan doses [46], another one [47] found differences in DLT rates at the same irinotecan dose level among wild-type, heterozygous, and homozygous patients, and one more other study [42] suggests the same irinotecan doses but combined with different capecitabine doses for each *UGT1A1* genotype.

As we can see (Table 3), the five studies finding lower MTDs for *UGT1A1*-heterozygous patients reported a mean 13.75% lower MTD in het patients compared to wild-types regardless of treatments and *UGT1A1* tested variants. Innocenti F. et al. [44] found 16.65% lower MTDs for *UGT1A1*-heterozygous patients compared to *UGT1A1*-wild-type patients, 16.12% lower in the trial by Toffoli G. et al. [49], at 18.75% by Zhu J. et al. [48], and 16.21% in the other trial by Toffoli G. et al. [51]. Also, Kim K.P. et al. [50] found a 9.1% lower MTD among *UGT1A1* heterozygous patients if we consider 330 mg/m^2^ the MTD for these patients, but this study reports that the MTD for these patients is higher than 330 mg/m^2^.

Thus, according to the results of the clinical trials found in this systematic review, the dose of irinotecan may be adjusted also in patients carrying a single mutated allele, particularly for the UGT1A1*28 and *6 variants. The results agree with the dose-adjustment recommendations for patients carrying two mutated *UGT1A1* alleles, which is to administer 70% of the usual dose, or what is the same, a 30% reduction in the initial dose of the treatment. Therefore, we suggest a 15% reduction in the initial irinotecan dose for patients carrying compared to UGT1A1*28 or *6 alleles, although the usefulness of this recommendation must be confirmed with the necessary studies.

It is also interesting that, actually, according to the information contained in PharmGKB [17] and as we can see in the included studies, UGT1A1*6 and *28 are the only *UGT1A1* alleles that have been associated with different MTDs, DLTs, and a response to irinotecan with a level of evidence of 1A. In contrast, there is no consensus regarding the *UGT1A1* variants referred to in the therapeutic guidelines based on PGx and/or irinotecan drug labels approved by the different agencies. Regarding ONYVIDE^®^, the EMA and FDA only refer to the UGT1A1*28 allele, and the FDA drug label of CAMPTOSAR^®^ refers to UGT1A1*6 and *28. ONIVYDE^®^ is pegylated liposomal irinotecan and CAMPTOSAR^®^ is irinotecan in the form of hydrochloride, but in the Phase I trials for both drugs (see Table 3) considering the *UGT1A1* genotype, different DLTs and MTDs are shown depending on the genotype of the patients.

On the other hand, the DPWG guidelines make special reference to UGT1A1*28 but consider other variants, including the UGT1A1*6 and *37 alleles, for the translation of the genotype into a UGT1A1 IM or PM phenotypes, and the SEFF guidelines refer to the UGT1A1*6, *28, *36, and *37 alleles, although they only give therapeutic recommendations for carriers of two mutated alleles among them (UGT1A1 PM phenotype). Obviously, both agencies provide enough evidence supporting their decision to include all these *UGT1A1* variants in their guidelines and, even, in the near future, irinotecan SmPC should be updated considering their conclusions. Results in this systematic review do not reject the decision of these agencies to provide therapeutic recommendations to irinotecan-treated patients based on *UGT1A1* variants different from UGT1A1*28 and *6, but we do suggest that UGT1A1 IM patients may be also dose adjusted.

Anyway, all of this is another example of the need to harmonize the criteria for inclusion of a genetic variant in existing PGx-dosing guidelines, as well as some form of PGx-based therapeutic recommendation in a drug’s SmPC.

## 5. Conclusions

Based on this systematic review, we conclude that the MTD of irinotecan is different among patients carrying none, one, or two *UGT1A1*-mutated alleles, considering UGT1A1*28 and/or *6.

Also, the *UGT1A1* genotype, considering the UGT1A1*28 and/or *6 alleles, is associated with ADE occurrence among irinotecan-treated patients, even if we compare *UGT1A1*-wild-type and heterozygous patients (UGT1A1*1/*28 or *1/*6 genotypes).

Based on this, we should conclude that different irinotecan doses may be recommended to patients carrying a *UGT1A1* heterozygous genotype compared to wild-type carriers, and a 15% dose reduction might be considered. On the other hand, this therapeutic recommendation must be tested in a Phase III clinical trial to confirm the efficacy of the treatment.

## Figures and Tables

**Table 1:** *UGT1A1* variants and their minor allele frequencies.

*UGT1A1* Allele	rsID				Enzyme Function ^^^
	rs887829	rs3064744	rs4148323	rs35350960	
*1	C	A(TA)7A	G	C	normal
*6			A		decreased
*27				A	decreased
*28		A(TA)8A			decreased
*36		A(TA)6A			increased
*37		A(TA)9A			decreased
*80	T				unknown
*80 + *28	T	A(TA)8A			decreased
*80 + *37	T	A(TA)9A			decreased
**Allele Frequency**					
IBS (1000 genomes)	28.04 (T)	-	0.00 (A)	0.00 (A)	
Europe (1000 genomes)	29.82 (T)	-	0.70 (A)	0.00 (A)	
Global (1000 Genomes)	35.40 (T)	-	3.43 (A)	0.28 (A)	
All populations (ALFA)	32.79 (T)	17.01	0.67 (A)	0.09 (A)	

^^^ According to the Clinical Pharmacogenetics Implementation Consortium (CPIC) function assignment. Allele definitions obtained from PharmGKB [17]. Allele frequency data obtained from the 1000 Genomes Project [18] and from the Allele frequency Aggregator (ALFA) [19]; rsID: reference single nucleotide polymorphism (SNP) ID; IBS: Iberian Peninsula population.

**Table 2:** Phase I clinical trials examining irinotecan or related active metabolites considering *UGT1A1* variants for dose tailoring.

Ref.	Phase	Population (Diagnosis)	Chemotherapy Scheme	Follow-up Cycles. (in DLT Study)	Primary Endpoint	Ethnicity ^1^	N	rs	Allele	MAF%	Genotype MM/Mm/mm
[42]	I	Multiple	CAPIRINOX	All (1)	Safety, efficacy, PK	White	50	rs3064744 (TA8)	*28	40.0	21/18/11
[43]	I	Metastatic GI cancer	Monotherapy	2 (2)	Safety, PK	Japan	82	rs3064744 (TA8)	*28	12.2	41/20/21 ^2^
								rs4148323	*6	25.6	
[44]	I	Multiple	Monotherapy	All (1)	Safety, efficacy, PK	White	68	rs3064744 (TA8)	*28	33.8	31/28/9
[45]	I–II	Locally advanced/metastatic GI cancer	FOLFIRABRAX	All (1)	Safety, efficacy	White	50	rs3064744 (TA8)	*28	34.0	23/20/7
[46]	I	Pancreatic adenocarcinoma	OXIRI	All (1)	Safety, efficacy, PK/PD	China	36	rs3064744 (TA8)	*28	9.7	18/17/1 ^4^
								rs4148323	*6	16.7	
[47]	I	Metastatic CRC	XELIRI	3y (1)	Safety, efficacy, PK	Korea	50	rs3064744 (TA8)	*28	12.0	23/20/7 ^3^
								rs4148323	*6	22.0	
[48]	I	Locally advanced rectal cancer	XELIRI	All (1)	Safety, efficacy	Asian	26	rs3064744 (TA8)	*28	-	15/11/exc
[49]	I	Metastatic CRC	FOLFIRI + Bevacizumab	All (1)	Safety, efficacy, PK	White	48	rs3064744 (TA8)	*28	-	25/23/exc
[50]	I	Metastatic CRC	FOLFIRI	All (1)	Safety, PK	Korea	43	rs3064744 (TA8)	*28	10.5	19/20/4 ^5^
								rs4148323	*6	22.1	
[51]	I	Metastatic CRC	FOLFIRI	All (1)	Safety, efficacy, PK	white	63	rs3064744 (TA8)	*28	25.4	35/24/4
[52]	I	Multiple	IHL-305	All (1)	Safety, efficacy, PK	White	37	rs3064744 (TA8)	*28	16.2	25/12/0
[53]	I	Multiple	NK012	All (1)	Safety, efficacy, PK	White	38	rs3064744 (TA8)	*28	34.2	19/12/7
[54]	I	Recurrent malignant glioma	nal-IRI	1 (1)	Safety, efficacy, PK	NS	34	rs3064744 (TA8)	*28	-	16/18/exc

Ref, reference; DLT, dose-Limiting toxicity; rs, reference single-nucleotide polymorphism (SNP); MAF, minor allele frequency; GI, gastrointestinal; CRC, colorectal cancer; CAPIRINOX, capecitabin, irinotecan, and oxaliplatin; FOLFIRABRAX, 5-fluorouracil, leucovorin, irinotecan, and nab-paclitaxel; OXIRI, oxaliplatin and capecitabine, and irinotecan; XELIRI, irinotecan and capecitabine; FOLFIRI, leucovorin, 5-fluorouracil, and irinotecan; IHL-305, pegylated liposome containing irinotecan; NK012, an SN-38-incorporating macromolecular polymeric micelle; Nal-IRI, irinotecan liposome injection; PK, pharmacokinetics; PD, pharmacodynamics; Exc, patients carrying an UGT1A1*28/*28 genotype were excluded in the study; NS, not stated. ^1^ It is reported the predominant ethnicity including more than the 80% of patients in each trial. ^2^ The mM (heterozygous) group includes *n* = 8 *1/*28, and *n* = 12 *1/*6 patients, and the MM (recessive homozygous) group includes *n* = 3 *28/*28, *n* = 12 *6/*6, and *n* = 6 *28/*6. ^3^ The mM (heterozygous) group includes *n* = 6 *1/*28 and *n* = 11 *1/*6, and the MM (recessive homozygous) group includes *n* = 1 *6/*28. ^4^ The mM (heterozygous) group includes *n* = 6 *1/*28 and *n* = 14 *1/*6, and the MM (recessive homozygous) group includes *n* = 3 *6/*6, *n* = 2 *28/*28, and *n* = 2 *6/*28. ^5^ The mM (heterozygous) group includes *n* = 6 *1/*28 and *n* = 14 *1/*6, and the MM (recessive homozygous) group includes *n* = 1 *1/*28, *n* = 2 *6/*6, and *n* = 1 *6/*28.

**Table 3:** Dose-limiting toxicities and maximum-tolerated doses reported in Phase I clinical trials considering the irinotecan–*UGT1A1* interaction.

Ref.	Scheme	*UGT1A1* Genotype: n	Irinotecan Dose mg/m^2^ (*n*)	DLT *n* (%)	MTD mg/m^2^	DLT Definition (Summarized)
[43]	Monotherapy	Wt: 40	150 (40)	1 (2.5)	>150	G4 neutropenia, G4 thrombocytopenia, febrile neutropenia (neutrophil count < 1000/mm^3^ and fever ≥ 38.5 °C), or G3 diarrhea.
		Het: 20	150 (16)	0 (0)	>150	
		Hom: 19	150 (16)	6 (37.5)	150	
[42]	CAPIRINOX	Wt: 21	175 (3)	2 (66.7)	150 ^^1^	G4 absolute neutrophil count > 5 days, G4 hemoglobin, platelet < 25,000/μL, serum creatinine ≥ 2× baseline, treatment delay >14 days, and sensory neuropathy ≥ G3. G3-4 non-hematologic toxicity. Inability to complete 10 days of prescribed dose of capecitabine during cycle one.
			150 ^^1^ (12)	5 (41.7)		
			150 ^^2^ (6)	0 (0)		
		Het: 18	150 ^^3^ (6)	2 (33.3)	150 ^^4^	
			150 ^^4^ (12)	0 (0)		
		Hom: 11	150 (2)	1 (50)	75 ^^4^	
			100 (5)	2 (40)		
			75 (6)	0 (0)		
[45]	FOLFIRABRAX	Wt: 23	180 (23)	5 (21.7)	-	Non-hematologic: ≥grade 3 events. Hematologic: G4 neutropenia lasting ≥ 5 days; G3-4 neutropenia with fever ≥ 38.5 °C and/or infection requiring antibiotics; G4 thrombocytopenia; and G3 thrombocytopenia accompanied by ≥ G2 hemorrhage. 14 days delay of cycle 1.
		Het: 19	135 (19)	1 (5.3)		
		Hom: 7	90 (7)	0 (0)		
[47]	XELIRI	Wt: 23	<350 (6)	0 (0)	380	G4 neutropenia > 5 days, febrile neutropenia, G4 thrombocytopenia or any other G3-4 non-hematological toxicity that did not improve after the institution of appropriate therapy, other toxicities that prevented completion of the prescribed dose of capecitabine during the first cycle, ADEs causing a delay > 2 weeks of the second cycle.
			350 (11)	2 (18.2)		
			380 (6)	2 (33.3)		
		Het: 20	<350 (10)	0 (0)	380	
			350 (8)	1 (12.5)		
			380 (2)	2 (100)		
		Hom: 7	200 (4)	0 (0)	240	
			240 (3)	2 (66.7)		
[46]	OXIRI	Wt: 18	-	-	75	Hematological: ≥ G3 neutropenia with infection and ≥3 thrombocytopenia with bleeding > 7 days. Non-hematological: ≥3 nonhematological toxicity other than untreated G3 diarrhea and nausea/vomiting, elevated alkaline phosphatase, and gamma glutamyl transferase levels.
		Het: 17			75	
		Hom: 1			50	
[44]	Monotherapy	Wt: 31	700 (9)	1 (11.1)	850	G4 neutropenia ≥ 4 days, ≥G3 neutropenia/day, ≥G3 febrile neutropenia, G4 anemia/thrombocytopenia, ≥G3 diarrhea, ≥G3 nonhematologic toxicity, or G4 nausea/vomiting.
			850 (16)	4 (25)		
			1000 (6)	2 (33.3)		
		Het: 28	700 (22)	5 (22.7)	700	
			850 (6)	4 (66.6)		
		Hom: 9	700 (6)	3 (50)	400	
			850 (3)	3 (100)		
[49]	FOLFIRI ^$1^	Wt: 24	260 (10)	1 (10)	310	≥G4 hematologic toxicity or ≥G3 non-hematologic toxicity despite maximal supportive measures (such as anti-diarrheal and anti-emetics) according to the NCI Common Terminology Criteria for Adverse Events (version 3.0).
			310 (10)	2 (20)		
			370 (4)	2 (50)		
		Het: 23	260 (10)	2 (20)	260	
			310 (10)	4 (40)		
			370 (3)	2 (66.7)		
[50]	FOLFIRI	Wt: 19	≤300 (13)	0 (0)	>330	G4 neutropenia > 5 days, febrile neutropenia; G4 thrombocytopenia, and any other ≥ G3 nonhematologic toxicities that did not improve to despite management therapies.
			330 (6)	1 (16.7)		
		Het: 20	<300 (17)	0 (0)	300	
			300 (3)	2 (66.7)		
		Hom: 4	150 (4)	-	150 ^$2^	
[52]	IHL-305	Wt + het: 53	≤105 ^A^ (12)	3 (25)	160 ^B^	G4 hematologic toxicity ≥5 days; G3-4 febrile neutropenia; G4 thrombocytopenia; ≥G3 non-hematologic toxicities; prolonged QTc > 500 ms; or any toxicity resulting in a treatment delay > 1 week.
			140 ^A^ (5)	2 (40)	80 ^A^	
			<160 ^B^ (28)	1 (3.6)		
			160 ^B^ (6)	1 (16.7)		
		Hom: 7	210 ^B^ (2)	2 (100)	-	
			NS (7)	1 (14.3)		
[53]	NK012	Wt + het: 29	≤21 (12)	0 (0)	28	G4 hematologic toxicity ≥ 5 days; G3-4 febrile neutropenia or G4 thrombocytopenia; ≥G3 non-hematologic toxicities; prolonged QTc > 500 ms (G3); any ADE resulting in a treatment delay beyond 1 week.
			28 (12)	1 (8.3)		
		Hom: 7	37 (5)	2 (40)		
			18.5 (7)	0 (0)	-	
[48]	XELIRI	Wt: 15	≤80 (12)	1 (8.3)	80	Hematologic G4 toxicities and non-hematologic G3 toxicities, with the exception of skin reactions and hand–foot syndromes.
			95 (3)	2 (66.7)		
		Het: 11	≤65 (9)	1 (11.1)	65	
			80 (2)	2 (100)		
[51]	FOLFIRI	Wt: 35	≤370 (32)	2 (6.3)	370	Hematologic G4 toxicity or nonhematologic G3-4 toxicity that developed or persisted despite supportive measures.
			420 (3)	2 (66.7)		
		Het: 24	≤310 (20)	1 (5)	310	
			370 (4)	2 (50)		
		Hom: 4	exc	-	-	
[54]	nal-IRI	Wt: 16	≤180 (13)	2 (15.4)	120	Hematological: ≥G3 thrombocytopenia > 5 days. G4 neutropenia > 5 days. G4 anemia of any duration. Non-hematological: ≥G3 toxicity except for G3 alopecia. Other: failure to recover from toxicities to be eligible for re-treatment within 35 days.
			240 (3)	2 (66.7)		
		Het: 18	≤120 (12)	1 (8.3)	150	
			150 (6)	1 (16.7)		

Ref, reference; ^^^ different capecitabine doses: ^1^ 1600 mg, ^2^ <1600 mg and >800 mg, ^3^ 800 mg, ^4^ 400 mg. ^A^ Each 14 days, ^B^ each 28 days. ^$1^ Plus bevacizumab. ^$2^ Dose not escalated based on previous studies. DLT: dose-limiting toxicities. MTD: maximum-tolerated dose. Wt: wild-type genotype. Het: heterozygous genotype. Hom: recessive homozygous genotype. GI: gastrointestinal. CRC: colorectal cancer. sLV5FU2: 5-fluorouracil and leucovorin. Exc: patients carrying an UGT1A1*28/*28 genotype were excluded from the study. CAPIRINOX: capecitabin, irinotecan, and oxaliplatin. FOLFIRABRAX: 5-Fluorouracil, leucovorin, irinotecan, and nab-paclitaxel. XELIRI: irinotecan and capecitabine. OXIRI: oxaliplatin and capecitabine, and irinotecan. FOLFIRI: leucovorin, 5-fluorouracil, and irinotecan; NCI: National Cancer Institute. IHL-305: pegylated liposome containing irinotecan. NK012: an SN-38 incorporating macromolecular polymeric micelle. Nal-IRI: irinotecan liposome injection. NS: not stated. PK: pharmacokinetics.

**Table 4:** Pharmacokinetic results based on *UGT1A1* genotype.

Reference	Genotype: n (Irinotecan Dose)	AUC (Mean ± sd)		AUC Ratio
		SN-38	SN-38G	
[43] AUC 0–24 h (ng × h/mL)	Wt: 46	264 ± 114	1266.8 ± 667.5	5.03 ± 2.25
	Het:16	279.6 ± 152.0	820.7 ± 378.7	3.25 ± 1.32
	Hom:16	509.8 ± 261.8	849.0 ± 561.9	1.85 ± 1.13
[42] AUC 0–48 h (ng × h/mL)	Wt: 9 (150)	291± 146	1124 ± 543	3.86 ± 3.72 *
	Het: 9 (150)	226 ± 100	876 ± 543	3.88 ± 5.43 *
	Hom: 3 (75)	135 ± 64	413 ± 84	3.06 ± 1.31 *
	Hom: 3 (100)	454 ± 81	865 ±433	1.91 ± 5.35 *
[47] AUC last/dose ng × h/mL/mg	Wt: 23	0.32 ± 0.23	2.76 ± 1.13	7.72 ± 3.90
	Het: 20	0.55 ± 0.37	3.77 ± 1.85	5.71 ± 3.00
	Hom: 7	0.97 ± 0.42	3.24 ± 1.19	2.72 ± 1.56
[46] (median range) AUC 0-inf ^^^	Wt: 14	0.9 (0.4−2.0)	5.2 (1.5−18.4)	6.3 (2.5−15.4)
	Het: 15	1.6 (0.6−4.0)	6.7 (2.4−22.2)	3.9 (2.14−11.9)
[44] AUC 0-Inf (mg × h/L)	Wt: 6 (400)	0.867 ± 0.708	1.37 ± 0.59	2.63 ± 1.95
	Wt: 3 (500)	1.196 ± 0.662	3.41 ± 2.19	2.65 ± 0.61
	Het/hom: 30 (700)	0.808 ± 0.655	2.76 ± 1.96	5.17 ± 5.06
	Het/hom: 22 (850)	0.868 ± 0.761	3.63 ± 2.15	5.43 ± 3.24
	Hom: 6 (1000)	0.665 ± 0.290	2.87 ± 0.81	4.81 ± 1.63
[50] AUC last/dose h × nmol/l/mg	Wt: 19	1.07 ± 0.44	6.63 ± 3.96	6.46 ± 2.73
	Het: 20	1.55 ± 0.61	6.83 ± 3.46	4.69 ± 1.82
	Hom: 4	1.67 ± 0.43	5.68 ± 1.55	3.49 ± 1.09

AUC, Area under the curve; sd, standard deviation; Wt, wild-type genotype; Het, heterozygous genotype; Hom, recessive homozygous genotype. * Result not reported in the original research and self-calculated (SN-38G/SN-38); ^^^ results reported as AUC 0-inf/dose/BSA (h/m^5^).

## Data Availability

No new data were created or analyzed in this study. Data sharing is not applicable to this article.

## Supplementary Materials

The following supporting information can be downloaded at: https://www.mdpi.com/article/10.3390/pharmaceutics17050542/s1, Table S1: Reported ADEs in included studies.

## Abbreviations

The following abbreviations are used in this manuscript:

ADE	Adverse drug event
ALFA	Allele frequency aggregator
APC	7-Ethyl-10-[4-N-(5-aminopentanoic ac-id)-1-piperidino]carbonyloxycamptothecin
AUC	Area under the curve
CAPIRINOX	Capecitabine, irinotecan, and oxaliplatin
CES1-2	Carboxylesterases type 1 and 2
Cmax	Maximum concentration
CPIC	Clinical Pharmacogenenetic Implementation Consortium
CPNDS	Canadian Pharmacogenomics Network for Drug Safety
DLT	Dose-limiting toxicity
DPWG	Dutch Pharmacogenetics Working Group
EMA	European Medicines Agency
EPAR	European Public Assessment Report
ESMO	European Society for Medical Oncology
FDA	Food and Drug Administration
G1-4	Grade 1-4
GI	Gastrointestinal
IBS	Iberian Peninsula
IHL-305	A pegylated liposome containing irinotecan
IM	Intermediate metabolizer
JCR	Journal Citation Report
MAF	Minor allele frequency
Nal-IRI	A irinotecan liposome injection
NCI	National Cancer Institute
NK012	An SN-38 (irinotecan active metabolite) incorporating macromolecular polymeric micelle
NM	Normal metabolizer
NOS	Newcastle–Ottawa quality assessment Scale
NPC	7-Ethyl-10-[4-(1-piperidino)-1-amino]carbonyloxycamptothecin
mCRC	Metastatic colorectal cancer
MTD	Maximum tolerated dose
FOLFIRABRAX	5-Fluourouracil (5-FU), irinotecan, and nab-paclitaxel
FOLFIRI	Leucovorin (folinic acid), 5-FU, and irinotecan
FOLFIRINOX	Leucovorin (folinic acid), 5-FU, irinotecan, and oxaliplatin
OXIRI	Oxaliplatin (O), chronomodulated capecitabine (X), irinotecan (IRI)
PGx	Pharmqacogenetic
PK	Pharmacokinetic
PM	Poor metabolizer
RNPGx	French National Network of Pharmacogenetics
rsID	Reference SNP ID
SEFF	*Sociedad Española de Farmacogenética y Farmacogenómica* (Spanish Society of Pharmacogenetics and Pharmacogenomics)
SmPC	Drug’s summary of product characteristics
SNP	Single nucleotide polymorphism
UGT1A1	Uridine diphosphate (UDP) glucuronosyltransferase family 1 member A1
XELIRI	Capecitabine and irinotecan (also known as CAPIRI)
