# Supplementary material for: Dose-Limiting Toxicities and the Maximum Tolerated Dose of Irinotecan Based on UGT1A1 Genotypes: A Systematic Review
Source: Pharmaceutics. 2025 Apr 22;17(5):542. doi: 10.3390/pharmaceutics17050542 (PMC12114900; doi:10.3390/pharmaceutics17050542)
Supplement: Supplementary file 1 [file pharmaceutics-17-00542-s001.zip › pharmaceutics-3479560-supplementary.pdf]

Table S1: Reported ADEs in included studies.

Reference	Genotype	Non-hematological				Hematological		
		**Diarrhea G3/G4-5**	**Vomiting or nausea G3/G4-5**	**Fatigue or asthenia G3/G4-5**	**Other G3/G4-5**	**Neutropenia G3/G4-5**	**Leukopenia G3/G4-5**	**Other G3/G4-5**
Satoh et al. (43)	*1/*1	0	0	1/0	1/0	4/0	1/0	0
	*1/*6	0	0	0	0	1/0	0	0
	*1/*28	0	0	0	0	2/0	0	0
	*6/*6	1/0	0	0	0	2/4	5/1	0
	*28/*28	0	0	0	0	2/1	2/0	0
	*28/*6	0	0	0	0	0/1	1/0	0
Goetz et al. (42)	*1/*1	5/1	9/0	0	0	0	0	0
	*1/*28	2/0	2/0	0	1/2	0	0	0
	*28/*28	2/0	4/0	2/0	4/0	1/1	0	3/0
Joshi S et al. (45)	*1/*1	3/0	3/0	0	0	0	0	1/0
	*1/*28	0	1/0	0	0	0	0	0
	*28/*28	0	0	0	0	0	0	0
Kim K et al. (47) ^	*1/*1	2	4	2	0	1	0	1
	*1/*6 - *1/*28	1	1	2	0	0	0	0
	*6/*6-*28/*28-*6/*28	0	2	0	0	3	0	1
Ng M et al. (46) ^	MTD group	2	0	1	2	5	0	2
	RP2D group	2	0	0	0	2	0	1
Innocenti F et al. (44)	*1/*1	3/1	0	0	0	0/3	0	3/1
	*1/*28	0	3/0	0	0	2/4	0	0/2
	*28/*28	1/0	0	0	1/0	0/1	0	1/0
Toffoli G et al. (49)	*1/*1	3/0	1/0	0	0	0	0	0/1
	*1/*28	2/0	0	0	2/0	0/5	0	0
Kim K et al. (50) ^	*1/*1	0	0	0	0	8	1	0
	*1/*6 - *1/*28	0	0	0	0	14	2	2
	*6/*6-*28/*28-*6/*28	0	0	0	0	0	0	
Infante J et al. (52) ^	*1/*1-*1/*28 (28-day dosing)	1	11	1	10	2	0	5
	*1/*1-*1/*28 (14-day dosing)	1	4	2	2	3	0	0
	*28/*28 (Both dosing schedules)	0	0	0	1	1	0	0

Burris H et al. (53) ^	*1/*1-*1/*28	0	0	0	0	14	9	10
	*28/*28	0	0	0	0	5	3	0
Zhu J et al. (48)	*1/*1	2/0	0	1/0	2/0	0/2	1/2	0
	*1/*28	2/0	0	1/0	0	1/0	2/0	0
Toffoli G et al. (51)	*1/*1	2/0	0	0/1	2/0	0	0	0/2
	*1/*28	1/0	1/0	2/0	1/0	0/1	0/1	0
Clarke J et al. (54)	*1/*1	4/0	0	1/0	2/0	0	0	0
	*1/*28	1/0	0	0	1/0	0	0	0

^ Grade 3 (G3) and Grade 4 (G4) adverse drug events (ADEs) reported together. HFS: Hand-Foot Syndrome
